# 

**Published:** 1883-12

**Authors:** 


### INDEX TO VOL. XXX.

**A**

A Coal Economizer.............. 20
A Russian Ice Palace........... 32
A Lay Sermon... ............. 38
A Natural Wish................ 61
Abscess of Antrum ............ 75
Analyses of Food.............. 81
Appropriate Foods.............. 86
A Caution to Young Mothers.... 98
Acid Drinks.......... ........ 131
Agnosticism in China.. ........ 134
An Ancient Street.............. 176
After-Dinner Speeches.. ....... 193
Annie Laurie ................ 198
Alaska Possibilities............. 15
American Pharmacy Abroad... 232
An Age......................... 239

**B**

Bacteria...... ........... .... 16
Birth Rate in France........... 25
Bad Temper and Insanity....... 57
Bad Air....................... 225
British Pastry................. 234
Bronchitis..................... 213

**C**

Change of Location for Health.. 6
Cure for Ivy Poisoning......... 9
Cure of Diabetes................ 11
Care of Children...... ....... 35
Cold Feet.......................59, 68
Croup........................... 62
Cremation in Europe........... 78
Colds.......................85, 226
Constipation.................... 91
Constipation and Piles.......... 218
Cataract of the Eye....... ..... 96
Cheerful People... ........... 118
Climate for Consumption....... 125
Care of the Feet............... 128
Constipation of Nervous Origin.. 169
Care of the Eyes...............
Cigarette Smoking.............. 153
Cancer.......................... 213

**D**

Diet........................... 8
Digestibility of Oysters ........ 9
Decay of Teeth................ 28
Dangerous Cosmetics........... 30
Dieting for Health.............. 56
Digestibility of Coffee and Sugar 76
Dangers of Phosphorus.......... 83
Driven Wells.... .............. 90
Dangers of the Cold Bath....... 95
Destruction of Forests..........
Diptheria and Nephritis......... 174
Deafness from Smoking......... 179

**E**

Eucalyptus Trees............... 76
Economy.................... ... 67

**F**

Forced Feeding in Phthisis...... 10
Facts Worth Knowing.......... 18
Feminine Deformity.... ....... 29
Facts Worth Remembering...... 36
Feeding Horses............ .... 41
Finger Hails................... 58
Feet and Shoes................ 93
Food Adulterations ........9[illegible], 142
First Appearance............... 162
Fat and Lean................... 207
Fever and Ague... ............ 208
Fig Paste....................... 230

**H**

How to Sleep.................... 2
Hot and Cold Drinks............ 13
Healthy Homes................. 22
Health Capacity................ 35
How to Get Pure Water......... 44
Human Health................. 46
Hereditary Disease............. 49
Hardening the Constitution..... 58
Health........................ 61
How to Apply Soda in Burns.... 78
How to be Young at Eighty..... 118
How Greasers Live............. 135
Hair and Races................ 143
Health Means Dollars........... 151
Habits of Ostriches............ 164
How a Man Walks.............. 198
Hot Water Cure................ 209
H. W. Beecher's Sermons....... 222

**I**

Improvement in Chimneys...... 14
In the Water................... 77
Inheritance.................... 124
Ice for Teething Children....... 195
Insidious Destroyers............ 211

**J**

John Hoyard Payne............. 159

**L**

Leave-Taking................... 140
Laws of Health................. 70

**M**

Migration of Fish.... ........ 21
Mushroom and its Poison........ 24
Meerschaum................... 27
Methods of Building up Health. 105
Measles....................... 125
Man and Insects................ 152
Made Useful................... 194

**N**

National Park.................. 136
New and Stale Bread .......... 156
Nutriment...................... 227
Neuralgia...................... 187
Nature of Consumption......... 49

**O**

Opium Smoking................ 176
Oldest Tree in the World........ 179
Our Readers.................... 205
Objects of Medication........... 19
Object of Exercise.............. 224

**P**

Physical Exercise.............. 26
Pneumonia.................... 33
Practical Knowledge............ 50
Popular Science................. 102
Pure Air and Pure Blood........ 107
Piles.......................... 115
Prolonged Internal Obstruction.. 155
Philosophy of Eating........... 228

**Q**

Quacks and Their Methods...... 89
Quinine in Whooping Cough.... 196

**R**

Rabies......................... 6
Remedy for Scrofula............ 12
Remedy for Catarrh............ 18
Railways of the World.......... 19
Rheumatism..................65, 188
Rice and Beans................ 88
Regulating the Bowels........ .. 127
Regulating Diet................ 145
Rules in Case of Fire........... 180

**S**

Simple Remedies............... 3
Some Large Lenses............. 17
Solidified Tea.................. 28
Scientific Notes... ............ 40
Seaweed as an Anti-Fat......... 40
Small-Pox..................... 52
Spring Diseases................ 54
Snake Bite and its Cure......... 74
Salted Meats................... 87
Slaves of Cooking Stoves........ 94
Sleep.......................... 123
Sour Stomach... ............... 126
Snakes and Snake Poison....... 132
Small Waists.......... ....... 139
Sick Headache...... .. ...... 146
Sleep.......................... 154
Seasonable Clothing............ 165
Spearing for Timber............ 170
Stammering................171–215
Swedish Women................ 175
Shooting Stars .. ............. 178
St. Vitus' Dance. . ............ 200

**T**

Typhoid Fever............... .. 12
To Cure a Cold................. 55
Tomato Flour.................. 135
Trees by the Roadside........... 144
The Old Churchyard............ 157
The Edible Fungi............... 157
The Streets of Venice....... ... 158
Trichinæ..... ................ 163
The Christian Era............... 171
The Enduring Violin............ 178
The Prince's Escape............ 190
Treatment of Goitre............ 192
The Planet Mars............... 197
The Home Doctor.............. 198
The Egyptian Sphynx.......... 203
The Rural Masses of China...... 231
The Weight of Great Men....... 237
The Efficacy of Vaccination..... 119

**V**

Ventilation........... ........ 1
Vulgar Habits.................. 6
Victims of Fashion............. 155

**W**

When to Eat Meat............. 17
Who are the Gypsies?........... 84
Weak Eyes ................... 93
Why no Rats in Ireland......... 138
Warnings ..................... 185
White Decay .................. 189
When to Take Medicine ........ 221
Weakly Children .... ......... 232

**Y**

Yellow Fever.................. 166

